# Supplementary material for: PCAS: An Integrated Tool for Multi-Dimensional Cancer Research Utilizing Clinical Proteomic Tumor Analysis Consortium Data
Source: Int J Mol Sci. 2024 Jun 18;25(12):6690. doi: 10.3390/ijms25126690 (PMC11203781; doi:10.3390/ijms25126690)
Supplement: Supplementary file 1 [file ijms-25-06690-s001.zip › Supplementary figure.pdf]

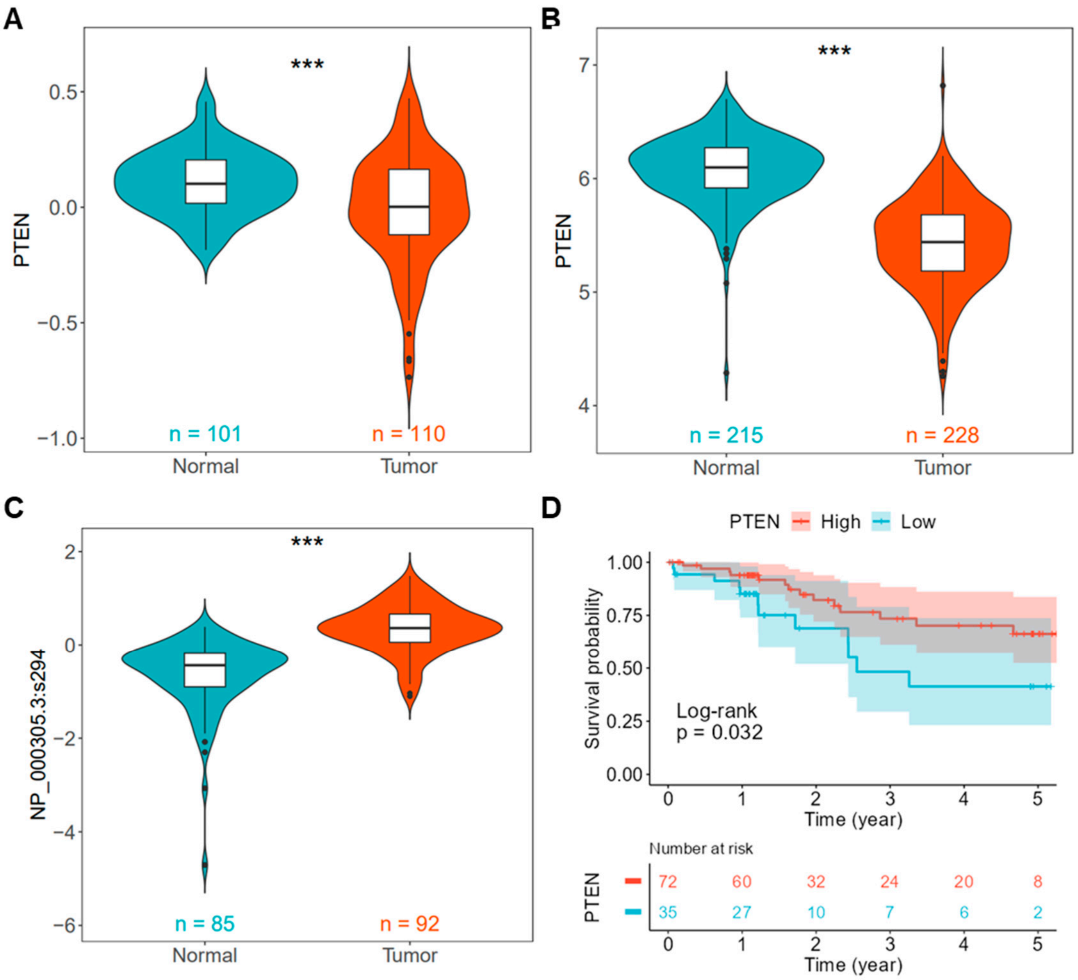

Figure S1. Analysis of *PTEN* expression and prognosis using the PCAS tool. (A) Differential protein expression of PTEN in tumor versus normal tissues in the LUAD_CPTAC_protein dataset. (B) Differential mRNA expression of *PTEN* in tumor versus normal tissues in the LUAD_CPTAC_mRNA dataset. (C) Differential expression of the PTEN phosphorylation site NP_000305.3s294 in tumor versus normal tissues in the LUAD_CPTAC_Phospho dataset. (E) Survival curves for high and low *PTEN* expression groups based on the LUAD_CPTAC_m RNA dataset. ***, $p < 0.001$ between two groups. CPTAC, Clinical Proteomic Tumor Analysis Consortium. LUAD, Lung adenocarcinoma.

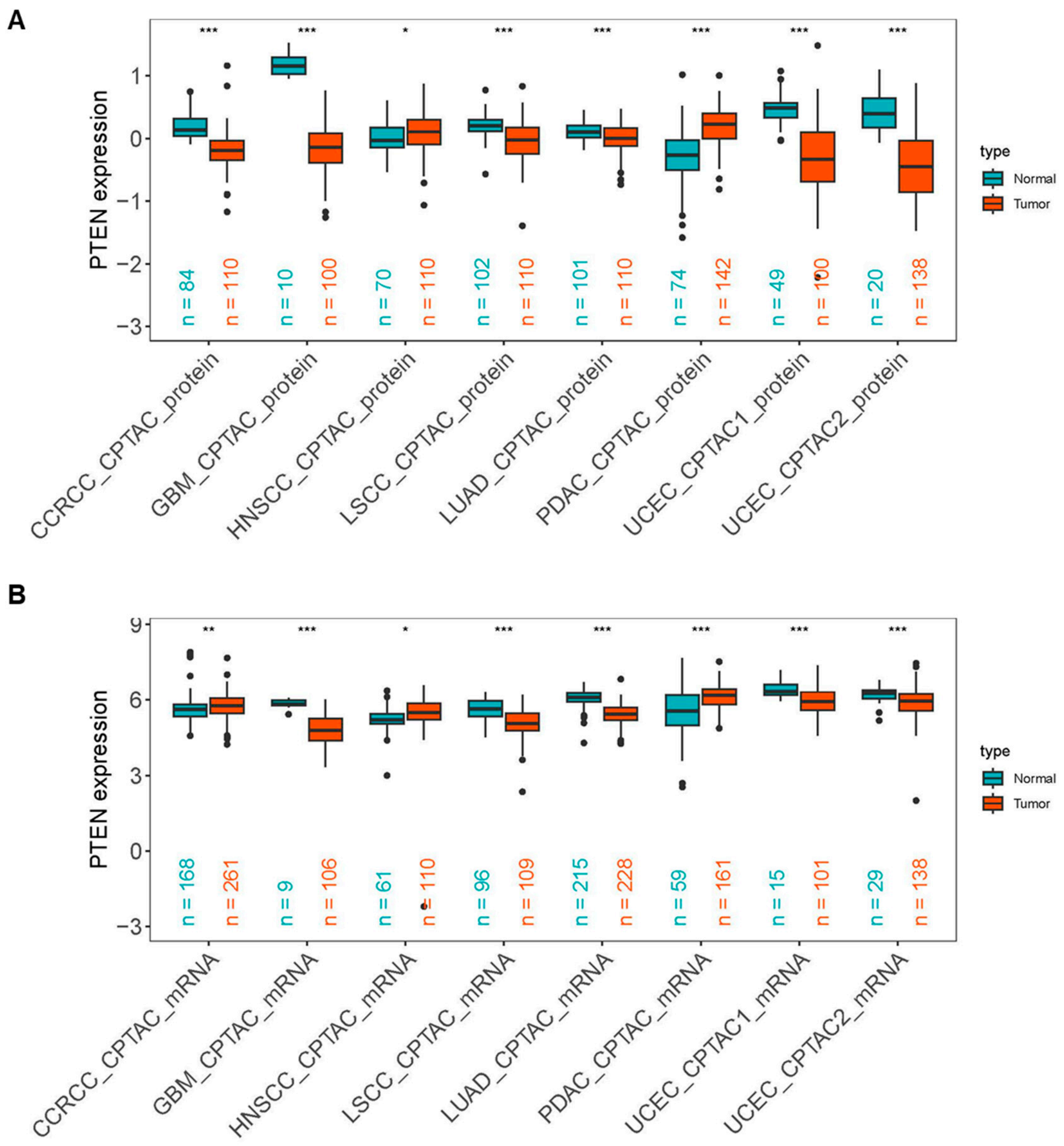

Figure S2. Analysis of *PTEN* expression across multiple datasets. (A) Differential RNA expression analysis of *PTEN* across multiple transcriptomic datasets. (B) Differential protein expression analysis of the PTEN across multiple proteomic datasets. *, $p < 0.05$ between two groups. **, $p < 0.01$ between two groups. ***, $p < 0.001$ between two groups. CCRCC: Clear Cell Renal Cell Carcinoma. GBM: Glioblastoma Multiforme. HNSCC: Head and Neck Squamous Cell Carcinoma. LSCC: Lung Squamous Cell Carcinoma. LUAD: Lung Adenocarcinoma. PDAC: Pancreatic Ductal Adenocarcinoma. UCEC: Uterine Corpus Endometrial Carcinoma. CPTAC, Clinical Proteomic Tumor Analysis Consortium.

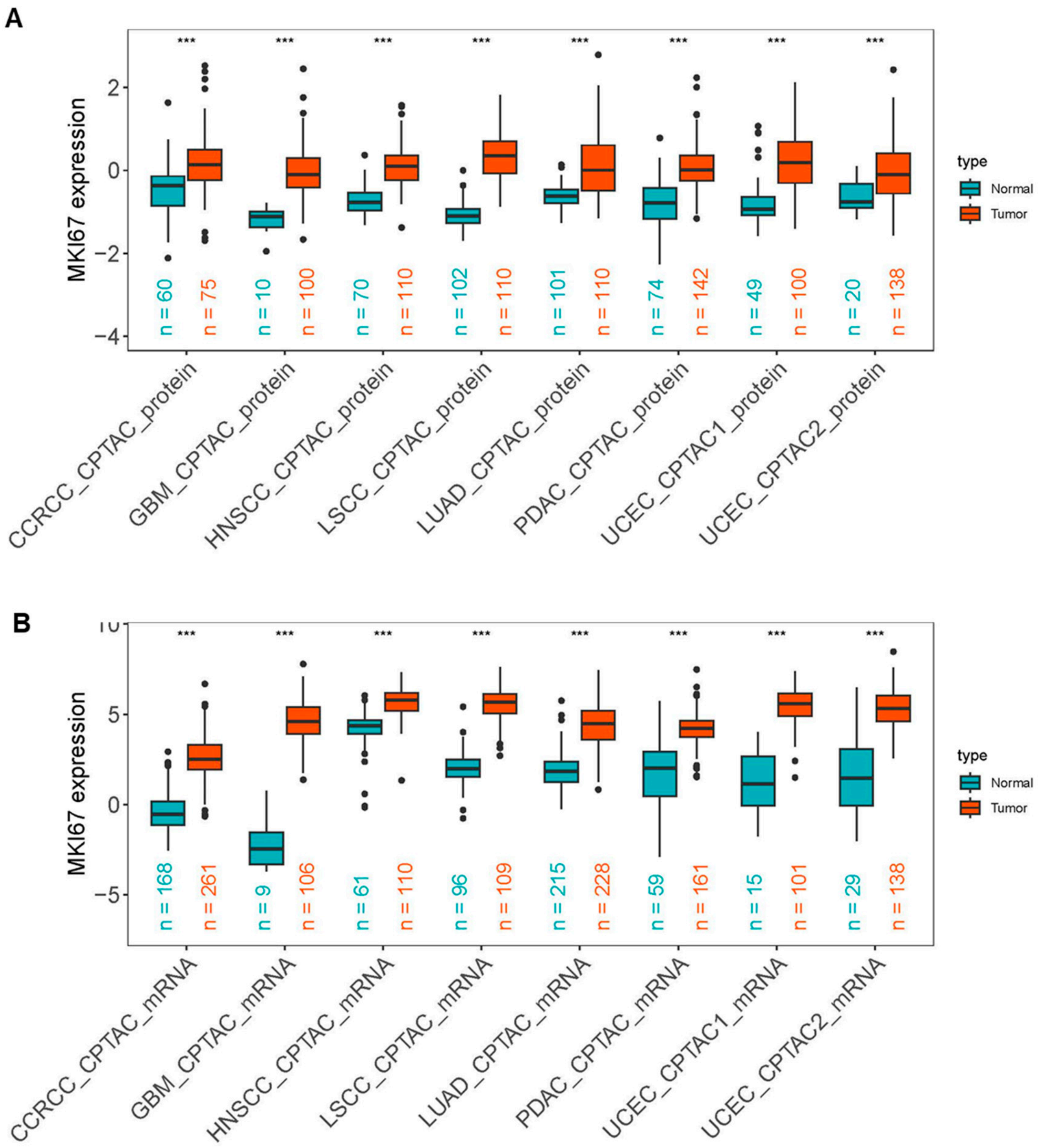

Figure S3. Analysis of *MKI67* expression across multiple datasets. (A) Differential RNA expression analysis of *MKI67* across multiple transcriptomic datasets. (B) Differential protein expression analysis of the MKI67 across multiple proteomic datasets. *, $p < 0.05$ between two groups. **, $p < 0.01$ between two groups. ***, $p < 0.001$ between two groups. CCRCC: Clear Cell Renal Cell Carcinoma. GBM: Glioblastoma Multiforme. HNSCC: Head and Neck Squamous Cell Carcinoma. LSCC: Lung Squamous Cell Carcinoma. LUAD: Lung Adenocarcinoma. PDAC: Pancreatic Ductal Adenocarcinoma. UCEC: Uterine Corpus Endometrial Carcinoma. CPTAC, Clinical Proteomic Tumor Analysis Consortium.
